# Characterisation of *Pasteurella multocida* isolates from pigs with pneumonia in Korea

**DOI:** 10.1186/s12917-019-1861-5

**Published:** 2019-04-25

**Authors:** Jongho Kim, Jong Wan Kim, Sang-Ik Oh, ByungJae So, Won-Il Kim, Ha-Young Kim

**Affiliations:** 1 Animal Disease Diagnostic Division, Animal and Plant Quarantine Agency, 177 Hyeoksin 8-ro, Gimcheon, Gyeongbuk 39660 Republic of Korea; 2 Laboratory of Immunology, College of Veterinary Medicine, Chonbuk National University, Iksan, 54596 Republic of Korea

**Keywords:** Antimicrobial resistance, Biovar, Capsular type, *Pasteurella multocida*, Virulence-associated gene

## Abstract

**Background:**

*Pasteurella multocida* is responsible for significant economic losses in pigs worldwide. In clinically diseased pigs, most *P. multocida* isolates are characterised as subspecies *multocida*, biovar 2 or 3 and capsular type A or D; however, there is little information regarding subspecies, biovars, and other capsular types of *P. multocida* isolates in Korea. Here, we provided information covering an extended time period regarding *P. multocida* in pigs with pneumonia in Korea using phenotypic and genotypic characterisations and data associated with the minimum inhibitory concentrations.

**Results:**

The overall prevalence of *P. multocida* between 2008 and 2016 was 16.8% (240/1430), with 85% of the *P. multocida* isolates (204/240) coinfected with other respiratory pathogens. Of the 240 isolates, 166 were included in this study; all of these *P. multocida* isolates were characterised as subspecies *multocida* and the most prevalent phenotypes were represented by biovar 3 (68.7%; *n* = 114) and capsular type A (69.9%; *n* = 116). Additionally, three capsular type F isolates were identified, with this representing the first report of such isolates in Korea. All biovar 1 and 2 isolates were capsular types F and A, respectively. The virulence-associated gene distribution was variable; all capsular type A and D isolates harboured *pmHAS* and *hsf-1*, respectively (*P* < 0.001), with type F (biovar 1) significantly correlated with *hsf-1* (*P* < 0.05) and *pfhA* (*P* < 0.01), biovar 2 highly associated with *pfhA* and *pmHAS*, and biovar 3 significantly correlated with *hsf-1*, *pmHAS*, and *hgbB* (*P* < 0.001), whereas biovar 13 was related only to *hgbB* (*P* < 0.05). The highest resistance rate was found to be to oxytetracycline (63.3%), followed by florfenicol (16.3%).

**Conclusions:**

*P. multocida* subspecies *multocida*, biovar 3, and capsular type A was the most prevalent isolate in this study, and our findings indicated the emergence of capsular type F in Korea. Moreover, prudent use of oxytetracycline and florfenicol is required because of the identified high resistance rates. Further studies are required for continuous monitoring of the antimicrobial resistance, prevalence, and epidemiological characterisation of *P. multocida*, and experimental infection models are needed to define the pathogenicity of capsular type F.

## Background

*Pasteurella multocida* (*P. multocida*) is a commensal and opportunistic pathogen of the oral, nasopharyngeal, and upper respiratory tract [1] and the causative agent of a wide range of infections leading to high economic impact [2]. In pigs, *P. multocida* is associated with progressive atrophic rhinitis (PAR), and together with other respiratory pathogens, plays a significant role in porcine respiratory disease complex (PRDC) [3–6]. *P. multocida* prevalence has been reported as 8.0% in diseased pigs with pneumonia or PAR in China, and from 10.3 to 15.6% in pigs with pneumonia in Korea. Additionally, *P. multocida* constitutes 15.6% of isolated respiratory pathogens in the United States [3, 5, 7, 8].

*P. multocida* can be divided into three subspecies (*multocida*, *septica*, and *gallicida*) and 13 biovars (1–10 and 12–14) based on carbohydrate fermentation and production of the ornithine decarboxylase (ODC) enzyme [9–11]. The majority of swine isolates are subspecies *multocida* and mostly assigned as biovars 2 or 3 [1, 10, 12, 13]. Additionally, five capsular types based on capsular antigens (A, B, D, E, and F) have been described in *P. multocida*, with capsular types A, B, D, and F recovered from swine [1, 14]. Capsular types A and D are most commonly cultured from pneumonic lungs and PAR, respectively, whereas capsular types B and F are rarely isolated from pigs [3, 14–16]. In Korea, numerous studies suggest that capsular type A is more prevalent in porcine pneumonia than type D [7, 8, 15]; however, limited information is available regarding subspecies, biovars, and other capsular types of *P. multocida* isolates in Korea.

*P. multocida* reportedly possesses various virulence factors that play a significant role in pasteurellosis and survival in the host environment [3, 17, 18]. Furthermore, there is a clear correlation between certain virulence factors and capsular types or biovars [1, 3]. The functions and target genes of these factors are detailed in Table 1 and include those encoding outer membrane and porin proteins (*oma87*, *ompH*, *plpB*, and *psl*), adhesins (*fimA*, *pfhA*, *ptfA*, *hsf-1*, and *hsf-2*), superoxide dismutases (*sodA* and *sodC*), iron-acquisition-related factors (*exbB*, *exbBD-tonB*, *fur*, *tbpA*, *hgbA*, and *hgbB*), neuraminidases (*nanB* and *nanH*), hyaluronidase (*pmHAS*), and toxin (*toxA*). Identifying which virulence factors are prevalent is necessary to predict the pathogenic behaviour of the isolates and select potential future vaccine candidates.

**Table 1:** Primers used for the detection of capsular types and virulence-associated genes in *Pasteurella multocida* isolates

Gene function	Target gene	Description	Sequence (5′ – 3′)	size (bp)	Reference
Capsule serotypes	*kmt1*	Identification of all *P. multocida* isolates	ATCCGCTATTTACCCAGTGG GCTGTAAACGAACTCGCCAC	460	[19]
	*hyaD*-*hyaC*	Serogroup A *cap* gene	TGCCAAAATCGCAGTGAG TTGCCATCATTGTCAGTG	1044	[20]
	*bcbD*	Serogroup B *cap* gene	CATTTATCCAAGCTCCACC GCCCGAGAGTTTCAATCC	760	[20]
	*dcbF*	Serogroup D *cap* gene	TTACAAAAGAAAGACTAGGAGCCC CATCTACCCACTCAACCATATCAG	657	[20]
	*ecbJ*	Serogroup E *cap* gene	TCCGCAGAAAATTATTGACTC GCTTGCTGCTTGATTTTGTC	511	[20]
	*fcbD*	Serogroup F *cap* gene	AATCGGAGAACGCAGAAATCAG TTCCGCCGTCAATTACTCTG	851	[20]
Outer membrane and porin proteins	*oma87*	Outer membrane protein 87	ATGAAAAAACTTTTAATTGCGAGC TGACTTGCGCAGTTGCATAAC	948	[17]
	*ompH*	Outer membrane protein H	CGCGTATGAAGGTTTAGGT TTTAGATTGTGCGTAGTCAAC	438	[17]
	*plpB*	Outer membrane protein	TTTGGTGGTGCGTATGTCTTCT AGTCACTTTAGATTGTGCGTAG	282	[18]
	*psl*	Porin protein	TCTGGATCCATGAAAAAACTAACTAAAGTA AAGGATCCTTAGTATGCTAACACAGGACGACG	470	[17]
Adhesins	*fimA*	Fimbriae	CCATCGGATCTAAACGACCTA AGTATTAGTTCCTGCGGGTG	866	[18]
	*pfhA*	Filamentous haemagglutinin	AGCTGATCAAGTGGTGAAC TGGTACATTGGTGAATGCTG	275	[21]
	*ptfA*	Fimbriae	TGTGGAATTCAGCATTTTAGTGTGTC TCATGAATTCTTATGCGCAAAATCCTGCTGG	488	[17]
	*hsf-1*	Autotransporter adhesion	TTGAGTCGGCTGTAGAGTTCG ACTCTTTAGCAGTGGGGACAACCTC	654	[18]
	*hsf-2*	Autotransporter adhesion	ACCGCAACCATGCTCTTAC TGACTGACATCGGCGGTAC	433	[18]
Superoxide dismutases	*sodA*	Superoxide dismutases	TACCAGAATTAGGCTACGC GAAACGGGTTGCTGCCGCT	361	[17]
	*sodC*	Superoxide dismutases	AGTTAGTAGCGGGGTTGGCA TGGTGCTGGGTGATCATCATG	235	[17]
Iron acquisition related factor	*exbB*	Iron regulated and acquisition factors	TTGGCTTGTGATTGAACGC TGCAGGAATGGCGACTAAA	283	[18]
	*exbBD-tonB*	Iron acquisition related factors	GGTGGTGATATTGATGCGGC GCATCATGCGTGCACGGTT	1144	[17]
	*fur*	Iron regulated and acquisition factors	GTTTACCGTGTATTAGACCA CATTACTACATTTGCCATAC	244	[18]
	*tbpA*	Iron acquisition related factor	TGGTTGGAAACGGTAAAGC TAACGTGTACGGAAAAGCC	728	[21]
	*hgbA*	Haemoglobin binding protein	TGGCGGATAGTCATCAAG CCAAAGAACCACTACCCA	419	[17]
	*hgbB*	Haemoglobin binding protein	TCATTGAGTACGGCTTGAC CTTACGTCAGTAACACTCG	499	[21]
Neuraminidases	*nanB*	Neuraminidases	GTCCTATAAAGTGACGCCGA ACAGCAAAGGAAGACTGTCC	554	[17]
	*nanH*	Neuraminidases	GAATATTTGGGCGGCAACA TTCTCGCCCTGTCATCACT	360	[17]
Hyaluronidase	*pmHAS*	Hyaluronidase	TCAATGTTTGCGATAGTCCGTTAG TGGCGAATGATCGGTGATAGA	430	[18]
Toxin	*toxA*	Dermonecrotic toxin	TCTTAGATGAGCGACAAGG GAATGCCACACCTCTATAG	846	[21]

Antimicrobial resistance in pathogenic bacteria from food animals and the environment has become a global public health issue. Although beta-lactams, trimethoprim combination, florfenicol, macrolides, and tetracyclines have been shown to be the best antimicrobials for treating PRDC [6], resistance to these antimicrobials has been detected previously in *P. multocida* in many countries [3, 22–24]. In Korea, *P. multocida* isolates from pigs are sensitive to most antimicrobial agents, including ampicillin, ceftiofur, tilmicosin, and enrofloxacin, other than tiamulin [7].

To the best of our knowledge, only short-term studies have been performed to characterise porcine *P. multocida* isolates in Korea. This long-term study was carried out to provide baseline information regarding a large collection of *P. multocida* isolates from clinically diseased pigs by determining the distribution and association of capsular types, biovars, extensive virulence-associated gene profiles, and antimicrobial-resistance patterns.

## Results

### Prevalence of *P. multocida* in porcine pneumonic lungs

In total, 240 *P. multocida* isolates (16.8%) were recovered (Table 2); *P. multocida* was the second most frequently isolated bacterial pathogen in this study. Most isolates (85.0%; 204/240) were detected simultaneously with other respiratory pathogens, such as porcine reproductive and respiratory syndrome virus (PRRSV; 61.3%), porcine circovirus type 2 (PCV2; 37.5%), or *Streptococcus suis* (*S. suis*; 20.0%). *Mycoplasma hyorhinis* (MHR), *Actinobacillus pleuropneumoniae* (APP), *Mycoplasma hyopneumoniae* (MHP), *Haemophilus parasuis* (HPS), *Trueperella pyogenes* (*T. pyogenes*), and swine influenza virus (SIV) were detected to a lesser extent (19.2, 14.2, 10.4, 10.0, 4.6, and 3.8%, respectively). Of the *P. multocida* isolates, 166 were included in this study.

### Subspecies, biovar, and capsular type determination

The distribution of biovars and capsular types among the studied *P. multocida* isolates is shown in Table 3. All 166 isolates were identified as *P. multocida* subspecies *multocida*, which produces acid from sorbitol and glucose but not from dulcitol, lactose, and maltose. Most ODC-producing isolates belonged to biovar 3 (68.7%), followed by biovars 2 (21.1%) and 1 (1.8%). Interestingly, 14 isolates (8.4%) displayed identical carbohydrate fermentation results to biovar 3, except for ODC activity, and were thus assigned to biovar 13. All biovar 1 and 2 isolates comprised capsular type F and A, respectively (*P* < 0.001), whereas biovar 3 isolates comprised capsular types A and D (*P* < 0.001), and biovar 13 comprised capsular types A and D (*P* > 0.05). Capsular type A (69.9%) isolates were the most prevalent, followed by types D (28.3%) and F (1.8%), with none of the isolates in this study identified as type B or E. Importantly, this is the first report of capsular type F/biovar 1 isolation since 2014 (Table 3).

### Distribution of virulence-associated genes

Results of polymerase chain reaction (PCR) analysis of 21 virulence-associated genes showed that all *P. multocida* isolates harboured 14 genes (*oma87*, *ompH*, *plpB*, *psl*, *fimA*, *hsf-2*, *sodA*, *sodC*, *exbB*, *ExbBD-tonB*, *fur*, *hgbA*, *nanB*, and *nanH*), whereas *tbpA* was absent. Notably, the distribution of *toxA* (5.4%), *pfhA* (22.9%), *hsf-1* (34.9%)*, pmHAS* (69.9%), *hgbB* (78.3%), and *ptfA* (99.4%) varied among the 166 *P. multocida* isolates; this information and the distribution of virulence-associated genes according to capsular type and biovar are presented in Table 4. All capsular type A isolates harboured *pmHAS* (*P* < 0.001), and all capsular type D isolates harboured *hsf-1* (*P* < 0.001), with most (97.9%) also harbouring *hgbB* (*P* < 0.001). Additionally, capsular type F (biovar 1) was significantly correlated with *pfhA* (*P* < 0.01) and *hsf-1* (*P* < 0.05). Notably, *toxA* was present in only 5.4% (*n* = 9) of *P. multocida* isolates, which mainly belonged to biovar 3 (*n* = 9) and capsular type A (*n* = 8) (Table 4). Biovar 2 was highly associated with *pfhA* and *pmHAS*, whereas biovar 3 was significantly correlated with *hsf-1*, *pmHAS*, and *hgbB* (*P* < 0.001; Table 4). Most biovar 13 isolates harboured *pmHAS* and *hgbB*. The distribution of virulence-associated gene profiles of *toxA, hgbB,* and *pfhA* in the different biovars is shown in Table 5. All biovar 1 and 2 isolates were *toxA*^−^*hgbB*^*+*^*pfhA*^*+*^ and *toxA*^−^*hgbB*^*−*^*pfhA*^*+*^, respectively (*P* < 0.001), with the *toxA*^−^*hgbB*^*+*^*pfhA*^*−*^ profile present in most biovar 3 (*P* < 0.001) and all biovar 13 (*P* < 0.05) isolates.

### Antimicrobial susceptibility

The antimicrobial-resistance patterns, cumulative minimum inhibitory concentrations (MICs), MIC_50_, and MIC_90_ of *P. multocida* isolates from diseased pigs are shown in Table 6. Of the 18 antimicrobials tested, isolates exhibited the highest level of resistance to oxytetracycline (63.3%), followed by florfenicol (16.3%), penicillin (9.0%), ampicillin (7.8%), trimethoprim-sulfamethoxazole (3.0%), enrofloxacin (2.4%), and tulathromycin (0.6%), whereas all isolates were susceptible to ceftiofur and tilmicosin. The MIC_90_ values of antimicrobials for which breakpoints had not been determined according to Clinical and Laboratory Standards Institute (CLSI) criteria were as follows: chlortetracycline (2 μg/mL), spectinomycin (32 μg/mL), clindamycin (16 μg/mL), danofloxacin (0.5 μg/mL), gentamicin (4 μg/mL), neomycin (16 μg/mL), sulfadimethoxine (≥512 μg/mL), tiamulin (32 μg/mL), and tylosin (32 μg/mL).

**Table 2:** Prevalence of respiratory pathogens and the frequency of *Pasteurella multocida* co-infection with other pathogens

	No. of sample	Bacteria^a^							Virus^b^			None^c^
		PM	SS	MHR	APP	HPS	MHP	TP	PRRSV	PCV2	SIV	
Total no. (%) of samples in which pathogens were detected	1430	240 (16.8)	251 (17.6)	199 (13.9)	130 (9.1)	110 (7.7)	96 (6.7)	47 (3.3)	715 (50.0)	456 (31.9)	49 (3.4)	323 (22.6)
Total no. (%) of sample co-infected with *P. multocida*	240	240 (100)	48 (20.0)	46 (19.2)	34 (14.2)	24 (10.0)	25 (10.4)	11 (4.6)	147 (61.3)	90 (37.5)	9 (3.8)	–

^a^*PM Pasteurella multocida*, *SS Streptococcus suis*, *MHR Mycoplasma hyorhinis*, *APP Actinobacillus pleuropneumoniae*, *HPS Haemophilus parasuis*, *MHP Mycoplasma hyopneumoniae*, *TP Trueperella pyogenes*

^b^*PRRSV* porcine reproductive and respiratory syndrome virus, *PCV2* porcine circovirus type 2, *SIV* swine influenza virus

^c^None, none of the respiratory pathogen was detected from pneumonic lungs

**Table 3:** Distribution of biovars and capsular types among *P. multocida* isolates from 2008 to 2016

Biovar	Capsular type	Total No. (%)	No. (%) of positive isolates within the following years								
			2008	2009	2010	2011	2012	2013	2014	2015	2016
Biovar 1	Cap F	3 (1.8) ^***^	0	0	0	0	0	0	2 (14.3)	1 (4.0)	0
Biovar 2	Cap A	35 (21.1) ^***^	1 (10.0)	0	8 (34.8)	2 (18.2)	4 (23.5)	8 (25.8)	3 (21.4)	5 (20.0)	4 (13.3)
Biovar 3	Cap A	68 (41.0) ^***^	6 (60.0)	2 (40.0)	8 (34.8)	2 (18.2)	3 (17.6)	14 (45.2)	6 (42.9)	15 (60.0)	12 (40.0)
Biovar 3	Cap D	46 (27.7) ^***^	3 (30.0)	2 (40.0)	4 (17.4)	4 (36.4)	7 (41.2)	9 (29.0)	3 (21.4)	3 (12.0)	11 (36.7)
Biovar 13	Cap A	13 (7.8)	0	0	3 (13.0)	3 (27.3)	3 (17.6)	0	0	1 (4.0)	3 (10.0)
Biovar 13	Cap D	1 (0.6)	0	1 (20.0)	0	0	0	0	0	0	0
Total		166	10	5	23	11	17	31	14	25	30

^***^P < 0.001

**Table 4:** Distribution of virulence-associated (VA) genes according to capsular type and biovar in 166 *P. multocida* isolates

VA genes^a^	No. (%) of VA genes within the following capsular types			No. (%) of VA genes within the following biovars				Total No. (% of 166)
	Type A (69.9%, *n* = 116)	Type D (28.3%, *n* = 47)	Type F (1.8%, *n* = 3)	Biovar 1 (1.8%, *n* = 3)	Biovar 2 (21.1%, *n* = 35)	Biovar 3 (68.7%, *n* = 114)	Biovar 13 (8.4%, *n* = 14)	
*toxA*	8 (6.9)	1 (2.1)	0	0	0	9 (7.9) ^*^	0	9 (5.4)
*pfhA*	35 (30.2)^***^	0	3 (100) ^**^	3 (100) ^**^	35 (100) ^***^	0	0	38 (22.9)
*hsf-1*	8 (6.9) ^***^	47 (100) ^***^	3 (100) ^*^	3 (100)^*^	0	54 (47.4) ^***^	1 (7.1) ^*^	58 (34.9)
*pmHAS*	116 (100) ^***^	0	0	0	35 (100) ^***^	68 (59.6) ^***^	13 (92.9)	116 (69.9)
*hgbB*	81 (69.8) ^***^	46 (97.9) ^***^	3 (100)	3 (100)	0	113 (99.1) ^***^	14 (100) ^*^	130 (78.3)
*ptfA*	116 (100)	46 (97.9)	3 (100)	3 (100)	35 (100)	113 (99.1)	14 (100)	165 (99.4)

^*^P < 0.05, ^**^P < 0.01, ^***^P < 0.001

^a^All isolates contained the following genes: *oma87, psl, ompH, sodA, sodC, ExbBD-tonB, hgbA, nanB, nanH, hsf-2, plpB, fur, fimA, exbB*. However, *tbpA* was not found in any of the isolates

**Table 5:** Distribution of the *toxA*, *hgbB*, and *pfhA* gene profiles according to biovars

Gene profile of *toxA/hgbB/pfhA*	No. (%) of gene profiles within the following biovars				Total (*n* = 166)
	Biovar 1 (*n* = 3)	Biovar 2 (*n* = 35)	Biovar 3 (*n* = 114)	Biovar 13 (*n* = 14)	
*toxA* ^*−*^ *hgbB* ^*+*^ *pfhA* ^*+*^	3 (100)^***^	0	0	0	3
*toxA* ^*−*^ *hgbB* ^*−*^ *pfhA* ^*+*^	0	35 (100)^***^	0	0	35
*toxA* ^*+*^ *hgbB* ^*+*^ *pfhA* ^*−*^	0	0	9 (7.9)^*^	0	9
*toxA* ^*−*^ *hgbB* ^*+*^ *pfhA* ^*−*^	0	0	104 (91.2)^***^	14 (100)^*^	118
*toxA* ^*−*^ *hgbB* ^*−*^ *pfhA* ^*−*^	0	0	1 (0.9)	0	1

^*^P < 0.05, ^***^ *P* < 0.001

**Table 6:**
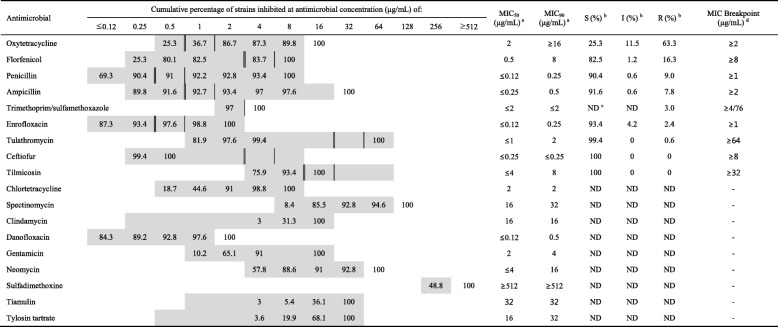
Antimicrobial susceptibility and cumulative percentage of *P. multocida* isolates (n = 166) for 18 antimicrobials

The grey zone represents the tested concentration range of each antimicrobial provided in the BOPO6F plate

Susceptibility and resistance breakpoints are indicated by double vertical (sensitive) and single vertical (resistant) lines according to the guidelines of each reference

^a^MIC_50_ and MIC_90_, concentrations at which the growth of 50 and 90%, respectively, of the isolates is inhibited

^b^*S* susceptible, *I* intermediate, *R* resistant

^c^*ND* not determined

^d^MIC breakpoints applied were those recommended by the Clinical and Laboratory Standards Institute (CLSI); trimethoprim/sulfamethoxazole interpretation was based on a previous study [25]

## Discussion

Our findings showed that *P. multocida* isolates were prevalent (16.8%) in pig farms and the second most frequently isolated bacterial pathogen from diseased pigs, following *S. suis* (17.6%). This was consistent with previous studies in Korea that reported the prevalence of *P. multocida* to be between 10 and 15.6% [7, 8]. The infections in this study comprised of a mix of *P. multocida* (85.0%) with other respiratory pathogens, particularly PRRSV (61.3%; *P* = 0.0001). Therefore, veterinary practitioners and surveillance stakeholders should consider coinfection with various pathogens that might exist in a given herd for PRDC control.

We characterised 166 *P. multocida* isolates by determining their subspecies, biovar, capsular type, virulence-associated genes, and MIC. To the best of our knowledge, this is the first report of biovar prevalence in Korea. All isolates belonged to subspecies *multocida*, and the most prevalent type was biovar 3 (68.7%), which is consistent with the results of previous studies of *P. multocida* recovered from pigs [1, 10, 13]. *P. multocida* biovar 1 is frequently isolated from poultry, but not pigs [1]. We found that the prevalence of biovar 13 was 8.4%, which is slightly higher than that in other countries, such as Australia (2.0%) and Hungary (4.8%) [10, 11]. In agreement with numerous previous studies, the dominant *P. multocida* capsular types recovered from pneumonic pig lungs were capsular types A (69.9%) and D (28.3%) [1, 15, 16, 26]. Additionally, capsular type B is the etiological cause of septicaemic pasteurellosis, whereas type F is rarely reported in pigs [1, 14]. Interestingly, capsular type F has been isolated in Korea post 2014, although at relatively low proportions (*n* = 3; 1.8%), the prevalence of which is consistent with that reported in other European studies [0.3% (Germany), 1.0% (UK), and 2.4% (Spain)] [1, 2, 16]. A recent Chinese experimental study indicated that pig-origin capsular type F isolates are associated with porcine pneumonia and exhibit high pathogenicity in pigs [27]. Additionally, we found that *P. multocida* capsular type F was the only relevant respiratory pathogen isolated from three growing pigs with moderate-to-severe suppurative bronchopneumonia with fibrous/fibrinous pleuritis. This represents the first report identifying capsular type F isolates in Korea; therefore, the pathogenic significance of type F in pigs needs to be elucidated.

Virulence genotyping is a useful typing method for molecular characterisation of bacterial pathogens and has been previously applied to *P. multocida* [1, 3]. Although *oma87*, *ompH*, *plpB*, *psl*, *fimA*, *hsf-2*, *sodA*, *sodC*, *exbB*, *ExbBD-tonB*, *fur*, *hgbA*, *nanB*, *nanH*, and *ptfA* were uniformly distributed among the isolates tested, none possessed *tbpA*, which agreed with the results of previous pig studies [1–3, 17]. The wide distribution of these genes indicates their importance for the survival of *P. multocida* within the host environment. Additionally, the virulence factors involved in cross-protection might constitute potential vaccine candidates, regardless of capsular type [3]. However, previous studies demonstrated that several non-uniformly distributed virulence-associated genes exhibit significant relatedness with specific capsular types [1, 3, 8, 17]. As shown in Table 4, all capsular type A and D isolates harboured *pmHAS* and *hsf-1*, respectively, and most type D isolates harboured *hgbB* (*P* < 0.001). In this study, capsular type F displayed virulence-associated gene profiles similar to those of capsular type D (*hsf-1*^+^*hgbB*^+^), except for *pfhA*. Previous studies reported *toxA* as clearly associated with type D [1, 3, 7, 17]; however, we found that only one of the 47 type D (2.1%) isolates and 6.9% of type A isolates harboured *toxA*. These results, however, are not significant because most of the isolates were from pneumonic lesions and not from turbinates with PAR. Similar to a previous report, distinct associations were observed between the virulence-associated gene profiles of *toxA, hgbB*, and *pfhA* and biovars, except for biovar 13 [1]. All biovar 1, 2, and 13 isolates exhibited *toxA*^*−*^*hgbB*^*+*^*pfhA*^*+*^ (*P* < 0.001), *toxA*^*−*^*hgbB*^*−*^*pfhA*^*+*^ (*P* < 0.001), and *toxA*^*−*^*hgbB*^*+*^*pfhA*^*−*^ (*P* < 0.05) profiles, respectively, and most biovar 3 isolates displayed a *toxA*^*−*^*hgbB*^*+*^*pfhA*^*−*^ profile (*P* < 0.001). Additionally, *toxA* was found only in biovar 3 isolates (*toxA*^*+*^*hgbB*^*+*^*pfhA*^*−*^; *P* < 0.05).

Swine diseases have become co-infected with immunosuppressive diseases, leading to antimicrobial treatment failure and frequent resistance occurrence. Treatment against *P. multocida* infections commonly includes broad-spectrum antimicrobials [3]. In this study, beta-lactams (penicillin, ampicillin, and ceftiofur), macrolides (tulathromycin and tilmicosin), and fluoroquinolone (enrofloxacin) were found to be more effective than oxytetracycline and florfenicol. Therefore, these agents are recommended as empirical antimicrobials for the treatment of *P. multocida* infection. Tetracycline resistance has previously been reported in *P. multocida* isolates worldwide [3, 6, 25, 28, 29]. Its prevalence in the present study was found to be 63.3%, which is similar to the prevalence in China (58.0%) and North America (53.4%) [3, 28] but higher than that in Australia (28.0%) and European countries (20.4%). Previous studies recommended the use of florfenicol for the treatment of infections caused by *P. multocida*, because florfenicol-resistance rates are very low (0–2%) in China, North America, Australia, and Europe [6, 25]; however, the present study showed a relatively higher resistance (16.3%). According to the Korea Animal Health Products Association, tetracyclines and florfenicol are the most commonly used antibiotics in Korean pig husbandry [30], with their frequent use reflected in the resistance rates in the present study. Based on the occurrence of high rates of tetracycline and florfenicol resistance, these antimicrobial agents should be used carefully and accompanied by susceptibility tests. Additionally, continuous surveillance of antimicrobial resistance in respiratory pathogens, including *P. multocida*, is required due to the increasing use of therapeutic antimicrobials and emergence of new resistant strains.

This study was conducted to determine the phenotypic and genotypic characteristics of swine *P. multocida* isolates in Korea. However, the collected samples cannot be representative of current *P. multocida* isolates in Korean swine farms, given that the number of isolates submitted annually varies, and the isolates used in this study originated from diagnostic samples with unknown antimicrobial-treatment history. However, a large-scale study for the characterisation of clinical lung samples of *P. multocida* isolates would sufficiently broaden the understanding of *P. multocida* as a respiratory pathogen.

## Conclusions

This represents a comprehensive report of *P. multocida* isolates in pigs in Korea. Our findings provide scientific information for further research, including development of vaccine candidates and guidelines for antimicrobial use in veterinary medicine. Moreover, the low discriminatory power of phenotypic characterisation limits the scope of adequate epidemiological information; therefore, different genotyping techniques using pulsed-field gel electrophoresis or multilocus sequence typing might be required to further elucidate the epidemiology of *P. multocida* and its genetic relatedness.

## Methods

### Bacterial isolation and identification

In total, 1430 lung samples were collected from pigs (suckling pigs, 9%; weaning pigs, 49%; growing-finishing pigs, 23%; and unknown, 19%) with pneumonic gross lesions from 514 farms nationwide between 2008 and 2016. All lung samples were submitted to the Animal and Plant Quarantine Agency for differential diagnosis of porcine respiratory diseases, including APP, HPS, *S. suis*, *T. pyogenes*, MHP, MHR, PRRSV, PCV2, and SIV. Following gross and histopathologic examination, samples were cultured on 5% sheep blood agar, chocolate agar (Asan Pharm. Co., Ltd., Seoul, Korea), and MacConkey agar (Becton Dickinson, Sparks, MD, USA) and then incubated aerobically at 37 °C for 48 h. Suspected mucoid and non-haemolytic colonies were subjected to Gram staining and biochemical identification using the VITEK II system (BioMérieux, Marcy l’Etoile, France). Identification was further confirmed by species-specific PCR assay for amplification of *kmt1* (Table 1) [19]. All *P. multocida* isolates were stored at − 80 °C until use to determine the subspecies, biovar, and capsular type. Previously reported methods were used to differentiate between *P. multocida* and other pathogens [3, 31, 32].

### Subspecies and biovar determination

The confirmed *P. multocida* isolates were classified into three subspecies (*multocida*, *septica*, and *gallicida*) based on sorbitol and dulcitol fermentation [9]. Additionally, isolates were assigned to one of the established biovars based on their ability to ferment carbohydrates (sorbitol, dulcitol, maltose, xylose, glucose, trehalose, lactose, and arabinose) and produce the ODC enzyme [10].

### PCR assay for capsular typing and virulence-associated gene detection

*P. multocida* isolates were inoculated into brain-heart infusion broth (Becton Dickinson) and cultured for 18 h. Genomic DNA was extracted using the QIAamp DNA mini kit (Qiagen, Hilden, Germany) according to manufacturer instructions. The capsular types of the isolates were determined by multiplex PCR using the capsule-specific primers shown in Table 1 [20]. PCR analysis of 21 virulence-associated genes, including *oma87*, *ompH*, *plpB*, *psl*, *fimA*, *pfhA*, *ptfA*, *hsf-1*, *hsf-2*, *sodA*, *sodC*, *exbB*, *exbBD-tonB*, *fur*, *tbpA*, *hgbA, hgbB*, *nanB*, *nanH*, *pmHAS*, and *toxA* (Table 1) [3, 17, 18, 21], was conducted as previously described. PCR amplification was performed using a Mastercycler ep Gradient S (Eppendorf, Hamburg, Germany), and amplified products were analysed with a capillary electrophoresis system (QIAxcel Advanced System; Qiagen). All tests were performed in duplicate in parallel with the relevant positive and negative controls.

### Antimicrobial-susceptibility testing

The MIC of all isolates (*n* = 166) was determined using the standard broth microdilution method with the Sensititre system (TREK Diagnostic System; Thermo Fisher Scientific, Cleveland, OH, USA) and commercially prepared 96-well antimicrobial testing plates containing 18 different agents (BOPO6F; TREK Diagnostic Systems). The following antimicrobials were tested: penicillin, ampicillin, ceftiofur, florfenicol, gentamicin, neomycin, chlortetracycline, oxytetracycline, clindamycin, enrofloxacin, danofloxacin, trimethoprim/sulfamethoxazole, sulfadimethoxine, spectinomycin, tulathromycin, tylosin tartrate, tilmicosin, and tiamulin. *Escherichia coli* ATCC 25922 was tested for quality control purposes. As shown in Table 6, the MICs were interpreted according to CLSI guidelines for oxytetracycline, florfenicol, penicillin, ampicillin, enrofloxacin, tulathromycin, ceftiofur, and tilmicosin or those of a previous study describing analysis of trimethoprim/sulfamethoxazole, for which CLSI breakpoints were not available [25, 33]. The overall MIC_50_ and MIC_90_ values (i.e., the lowest concentrations at which growth was inhibited by 50 and 90%, respectively) for each antimicrobial were determined for all isolates.

### Statistical analysis

Statistical testing was performed using GraphPad Prism (v5.01; GraphPad Software, San Diego, CA, USA) and SPSS (v22.0; IBM Corp., Armonk, NY, USA). Pearson’s chi-squared and Fisher’s exact tests were used to assess associations among capsular types, biovars, and virulence-associated genes. A *P* < 0.05 was considered statistically significant.

## Abbreviations

APP: *Actinobacillus pleuropneumoniae*
CLSI: Clinical and Laboratory Standards Institute
HPS: *Haemophilus parasuis*
MHP: *Mycoplasma hyopneumoniae*
MHR: *Mycoplasma hyorhinis*
MIC: Minimum inhibitory concentration
ODC: Ornithine decarboxylase
*P. multocida*: *Pasteurella multocida*
PAR: Progressive atrophic rhinitis
PCR: Polymerase chain reaction
PCV2: Porcine circovirus type 2
PRDC: Porcine respiratory disease complex
PRRSV: Porcine reproductive and respiratory syndrome virus
*S. suis*: *Streptococcus suis*
SIV: Swine influenza virus
*T. pyogenes*: *Trueperella pyogenes*
